# Correlation between renal distribution of leptospires during the acute phase and chronic renal dysfunction in a hamster model of infection with *Leptospira interrogans*

**DOI:** 10.1371/journal.pntd.0009410

**Published:** 2021-06-18

**Authors:** Tsukasa Maruoka, Yasuhiko Nikaido, Satoshi Miyahara, Eisuke Katafuchi, Yoshinori Inamasu, Midori Ogawa, Kazumasa Fukuda, Toshiyuki Nakayama, Takafumi Horishita, Mitsumasa Saito

**Affiliations:** 1 Department of Microbiology, School of Medicine, University of Occupational and Environmental Health, Kitakyushu, Japan; 2 Department of Anesthesiology, School of Medicine, University of Occupational and Environmental Health, Kitakyushu, Japan; 3 Department of Respiratory Medicine, School of Medicine, University of Occupational and Environmental Health, Kitakyushu, Japan; 4 Department of Pathology, School of Medicine, University of Occupational and Environmental Health, Kitakyushu, Japan; 5 Third Department of Internal Medicine, University of Occupational and Environmental Health, Kitakyushu, Japan; Tangen Biosciences, UNITED STATES

## Abstract

**Background:**

Leptospirosis has been described as a biphasic disease consisting of hematogenous dissemination to major organs in the acute phase and asymptomatic renal colonization in the chronic phase. Several observational studies have suggested an association between leptospirosis and chronic kidney disease (CKD). We investigated the dynamics of leptospires and histopathological changes in the kidney to understand the relationship between them, and also investigated the extent of renal dysfunction in the acute and chronic phases of leptospirosis using a hamster model.

**Findings:**

Hamsters (n = 68) were subcutaneously infected with 1 × 10^4^ cells of the *Leptospira interrogans* serovar Manilae strain UP-MMC-SM. A total of 53 infected hamsters developed fatal acute leptospirosis, and the remaining 15 hamsters recovered from the acute phase, 13 of which showed *Leptospira* colonization in the kidneys in the chronic phase. Five asymptomatic hamsters also had renal colonization in the chronic phase. Immunofluorescence staining showed that leptospires were locally distributed in the renal interstitium in the early acute phase and then spread continuously into the surrounding interstitium. The kidneys of the surviving hamsters in the chronic phase showed patchy lesions of atrophic tubules, a finding of chronic tubulointerstitial nephritis, which were substantially consistent with the distribution of leptospires in the renal interstitium. The degree of atrophic tubules in kidney sections correlated statistically with the serum creatinine level in the chronic phase (rs = 0.78, p = 0.01).

**Conclusion:**

Subcutaneous infection with pathogenic leptospires could cause acute death or chronic leptospirosis in hamsters after surviving the acute phase. We suggest that the renal distribution of leptospires during the acute phase probably affected the extent of tubular atrophy, leading to CKD.

## Author summary

Leptospirosis is the most widespread zoonotic disease, particularly in tropical and subtropical regions. Leptospires are transmitted to humans via contaminated urine, soil and water from carrier animals such as rodents. Human leptospirosis is mostly asymptomatic or a mild acute febrile illness, but some patients progress to Weil’s disease, characterized by kidney failure, jaundice, and pulmonary hemorrhage, and some of them die. To understand pathogen-host interactions in the kidney, a major target of leptospirosis, the authors collected kidneys and blood samples from the acute to chronic phase using a subcutaneously infected hamster model to compare the renal distribution of *Leptospira* with histopathological findings and renal function. The results demonstrated that the increase in antibody titers in the acute phase affected the outcome in the natural course of acute leptospirosis, and that the distribution of leptospires in the kidney during the acute phase may affect the decline of renal function during the chronic phase. It is possible that leptospirosis is one of the causes of unexplained renal dysfunction in tropical and subtropical regions.

## Introduction

Leptospirosis caused by pathogenic *Leptospira* is the most widespread zoonotic disease worldwide, especially in tropical and subtropical regions. In some carrier animals, such as rodents, leptospires colonize the proximal tubules of the kidney and are excreted in the urine. Leptospires are transmitted to humans via urine from infected animals, contaminated soil and water [1–3].

Human leptospirosis is mostly asymptomatic or a mild acute febrile illness characterized by flu-like symptoms, but some patients progress to Weil’s disease, a severe multiple organ failure, and some of them die. The kidneys, lungs, and liver are the major organs affected by severe leptospirosis [1–3]. The WHO reported that the median incidences of acute kidney injury and lung injury in leptospirosis were 36% and 17%, with median mortality rates of 12% and 25%, respectively [4].

*Leptospira* is a thin, helical spirochete, and it has been suggested that rotation of the inner flagellum and consequent corkscrew motility may play a role in the pathogenesis of infection [5]. The clinical course of severe leptospirosis is biphasic, beginning with a septicemic phase of about 1 week, followed by an immune phase characterized by antibody production and urinary shedding. First, leptospires penetrate the host percutaneously through wounds in the skin and mucous membranes. After that, they rapidly reach organs via hematogenous dissemination. During the immune phase, leptospires are eliminated from all organs except the kidney [1,6]. In fact, epidemiological studies in areas with a high prevalence of leptospirosis have reported that *Leptospira* forms asymptomatic renal colonization and is excreted in the urine by some people as well as reservoir animals [7].

The relationship between leptospirosis and chronic kidney disease (CKD) has attracted attention in recent years [8,9]. Two observational studies showed that past leptospirosis was associated with elevated estimated glomerular filtration rate (eGFR) and other sensitive renal biomarkers [10,11]. In a study of long-term renal function outcomes in acute kidney injury caused by leptospirosis, 9% of patients treated with antibiotics had abnormal renal function consistent with early CKD. Kidney biopsies of two CKD patients showed mild patchy interstitial lymphocyte infiltrate, tubular atrophy and interstitial fibrosis [12]. The severity and duration of renal dysfunction in leptospirosis vary greatly from case to case, but the factors that make these differences are not clearly understood.

The aim of this study was to understand the relationship between leptospirosis and chronic renal dysfunction using a hamster model. Hamsters, which are commonly used as animal models of severe human leptospirosis, are known to be chronic carriers if they survive the experimental infection without acute death. In a previous study, hamster models showed that chronic *Leptospira* infection results in tubulointerstitial nephritis and internal fibrosis [13,14]. The routes of infection have also been reported to affect the kinetics of hematogenous dissemination, kidney colonization, and inflammation [15–18]. Hamsters were selected as the experimental animals in this study, and subcutaneous injection, similar to a natural infection route, was selected as the infection route. We examined the dynamics of leptospires and histopathological changes in the kidneys, renal function, and anti-*Leptospira interrogans* serovar Manilae antibody titers from the acute phase to the chronic phase in the hamsters subcutaneously infected with pathogenic leptospires.

In the present study, we demonstrate that the increase in antibody titers in the acute phase influences the outcome in untreated acute leptospirosis, and suggest that the distribution of leptospires in the kidney during the acute phase (septicemic phase) can influence the decline in renal function during the chronic phase (after immunization).

## Materials and methods

### Animal ethics statement

The experiment was approved by the Animal Experiment Ethics Committee of the University of Occupational and Environmental Health, Japan (License No. AE 15–019). The experiments were conducted under the conditions stated in the Animal Experiment Regulations of the University of Occupational and Environmental Health, Japan and the Japanese Government’s Law and Notice.

### *Leptospira* strain, culture and storage

*Leptospira interrogans* serovar Manilae strain UP-MMC-SM (also known as L495 and UP-MMC-NIID) isolated from the blood of a patient with severe leptospirosis [19–22] was used in this study. The strain was cultured in Korthof’s medium and was grown to confluency at 30°C without shaking after in vivo passage. The strain was stored at room temperature in a dark place after reaching a stationary phase. The storage period was defined as the period after the last in vitro passage. In other words, in vitro passage was not performed during the storage period. *Leptospira* injected in this study varied in culture period (storage period) from one week to seven months. The virulence of the strain was maintained through monthly animal passage using hamsters. *Leptospira* used in the infection experiment was a low-passage strain with less than three in vitro passages. Motile bacterial cell counts were measured by dark-field microscopy using a Thoma counting chamber.

### Experimental infections and animals

Male Golden Syrian hamsters aged four to six weeks (Japan SLC, Inc., Shizuoka, Japan) were used as experimental animals. The culture of strain UP-MMC-SM was diluted with sterile Phosphate Buffered Saline (PBS) by 1 × 10^5^ leptospires / mL, and then 100 μL was injected subcutaneously into the right inguinal region of hamsters. The physical condition and body weight of the infected hamsters were monitored daily until 56 days post infection (dpi). The acute phase was defined as up to 13 dpi, and the chronic phase was defined as 14 dpi or later in this study. The moribund hamsters were euthanized in the acute phase. Of the hamsters that survived the acute phase, two to six were euthanized on each of 16, 22, 28, and 56 dpi in the chronic phase. The hamsters were anesthetized with sevoflurane, and then their thoraxes were opened. After the blood was collected by an incision in the right atrium, the hamsters were perfused from the left ventricle with 30 mL saline containing 0.5% heparin. Both of the kidneys, the right lung, the liver (Segment 4), and the spleen of the hamsters were collected. Urine was collected by bladder puncture.

### Measurement of the number of viable leptospires in organs, blood and urine

The right kidney, right lung, liver (Segment 4), and spleen collected from infected hamsters were homogenized in 2 mL of Korthof’s medium containing STAFF (sulfamethoxazole, trimethoprim, amphotericin B, fosfomycin and 5-fluorouracil). Twenty microliters of blood or urine were transferred to 980 μL of Korthof’s medium containing STAFF, respectively. The number of leptospires in the blood, urine, and each homogenized organ was calculated by the limiting dilution culture method on 96-well plates [22,23]. The plates were incubated at 30°C for 28 days, and the presence of leptospires was observed with a dark-field microscope.

### Histology and staining

The left kidneys recovered from the hamsters were fixed with 4% paraformaldehyde and 0.1% glutaraldehyde for more than 7 days. The fixed kidneys were sectioned into 4 μm slices with a microtome after paraffin embedding. Serial sections were deparaffinized, followed by hematoxylin and eosin staining (H&E staining), Periodic Acid Schiff staining (PAS staining), and immunofluorescence staining, as will be described later.

### Pathological scoring

All the kidney sections were pathologically examined by pathologists blinded to the experimental conditions. Inflammatory cell infiltration was defined as an accumulation of more than 50 inflammatory cells, and the number of spots with inflammatory cell infiltration in the renal cortex of each H&E-stained kidney section was counted to assess inflammatory changes. To evaluate the degree of scarred area, each kidney section stained with PAS was scored using a semiquantitative measure of the area of the atrophic tubules area as follows: 0 –no or few lesions; 1—atrophic tubules covering less than 25% of the area of the renal cortex; 2—atrophic tubules covering 25% to less than 50% of the area of the renal cortex; and 3—atrophic tubules covering more than 50% of the area of the renal cortex.

### Immunofluorescent staining

Deparaffinized left kidney sections were blocked with 3% bovine serum albumin in PBS (blocking buffer) at room temperature for 15 min, washed with PBS, and incubated overnight at 4°C with rabbit anti-*Leptospira interrogans* serovar Manilae antiserum (1: 200) as the primary antibody [22–25]. Sections were washed with PBS and incubated for 3 hours at room temperature with a goat anti-rabbit IgG antibody labeled with Alexa Fluor 488 (1: 500–1000; Life Technologies Co., USA) as a secondary antibody. After washing with PBS, the sections were incubated with 4’,6-diamidino-2-phenylindole (DAPI) (5μg/mL; Life Technologies Co., USA) for 10 minutes. After final washing with PBS, SlowFade Gold antifade (Life Technologies Co., USA) was applied to the sections on a slide glass, covered with a glass slide, and observed by virtual slide system VS120-L100-FL (Olympus, Japan).

### Microscopic agglutination test (MAT) and serum creatinine level measurement

Blood was collected from the ophthalmic venous plexus after anesthesia with sevoflurane in 22 infected hamsters that began to lose weight in the acute phase. Blood from euthanized hamsters was collected by an incision in the right atrium prior to the perfusions mentioned above. The blood samples were centrifuged twice at 1,000 ×g for 5 minutes to separate the serum. MAT was performed using *Leptospira interrogans* serovar Manilae strain UP-MMC-SM, twice for each serum sample, according to the WHO manual [26,27]. Inactivated serum that agglutinated more than 50% of live leptospires was determined to be MAT-positive. Serum dilutions ranged from 1:20 to 1:20480. A titer of 1:160 or more was considered as possible. The serum was stored at -20°C, and serum creatinine levels were measured later in a laboratory of Fujifilm VET Systems in Japan.

### The dynamics of the leptospires in moribund hamsters during the acute phase

1 × 10^4^ leptospires of strain UP-MMC-SM, cultured for 7 days in Korthof’s medium, were subcutaneously injected into the right inguinal region of 14 hamsters and observed daily. Two hamsters were also euthanized on each of 0, 2, 4, 6, 7, 8, and 9 dpi, and organs, blood, and urine were collected from each of them to observe the dynamics of the leptospires during the acute phase.

### Experimental infection of leptospires stored for a long period

Each leptospire suspension containing 1 × 10^4^ leptospires of strain UP-MMC-SM, stored for a long period (i.e. 1, 2, 3, 4, or 7 months) in Korthof’s medium, was subcutaneously injected into the right inguinal region of 5 hamsters and observed daily. The hamsters that survived the acute phase were euthanized on 16, 22, 28, and 56 dpi, and organs, blood, and urine were collected from each of them.

### Pathogenicity examination of leptospires excreted in the urine of surviving hamsters in the chronic phase of leptospirosis

Three hamsters that survived the acute phase were euthanized on 28 dpi, and urine was collected from each of them by bladder puncture. The urine was cultured in Korthof’s medium containing STAFF for 7 days at 30°C. Three cultures of *Leptospira* isolates obtained from the urine were diluted with PBS, and 1 × 10^4^ leptospires were injected subcutaneously into 5 hamsters each.

### Statistical analysis

Statistical analysis was performed using SPSS Statistics Base 25 (IBM, USA). The Mann-Whitney U test was used to compare the serum creatinine values between the moribund and the surviving hamsters in the acute phase. The Kruskal-Wallis test was used for comparisons of the serum creatinine values among the following 3 groups: the high atrophy tubules score group, the low atrophy tubules score group, and uninfected group. Spearman’s rank correlation coefficient was used to examine the correlation between the serum creatinine level and the following four factors: atrophic tubules score, number of spots with inflammatory cell infiltration, number of urinary leptospires, and number of renal leptospires. The significance level was set at less than 5%.

## Results

### Survival and positive kidney culture rates in hamsters subcutaneously infected with strain UP-MMC-SM

**Fig 1:**
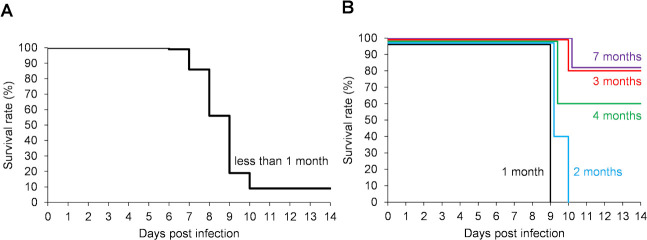
Survival (Kaplan-Meier curve) in the acute phase of hamsters subcutaneously infected with *L*. *interrogans*. Groups of four- to six-week-old male Golden Syrian hamsters were subcutaneously infected with 1 × 10^4^ cells of *L*. *interrogans* serovar Manilae strain UP-MMC-SM. The leptospires used for injection were cultured for (A) less than 1 month and (B) 1 (black), 2 (blue), 3 (red), 4 (green), and 7 (purple) months in Korthof’s medium after in vivo passage. A, n = 43 hamsters; B, n = 5 hamsters per group.

**Fig 2:**
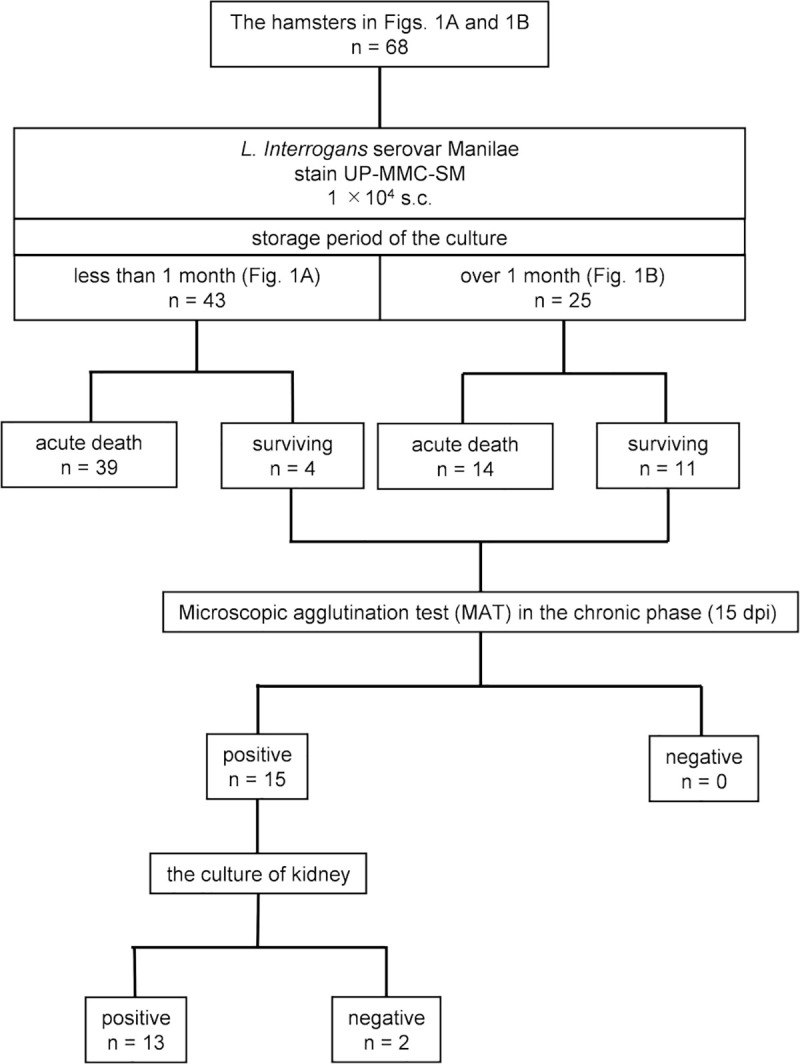
Microscopic agglutination test (MAT) and kidney cultures of surviving hamsters in the chronic phase. Fig 2 shows the outcomes of 43 hamsters (Fig 1A) and 25 hamsters (Fig 1B) and the results of MAT and kidney culture in those surviving hamsters.

Hamsters were infected subcutaneously with 1 × 10^4^ leptospires of strain UP-MMC-SM cultured in Korthof’s medium for less than 1 month (late log phase or stationary phase) after in vivo passage. Some infected hamsters began to be moribund on 6 dpi, the maximum number of moribund was on 9 dpi, and there were none after 11 dpi (Fig 1A). The survival rate of hamsters under this experimental condition was 9.3% (4/43) (Fig 1A). All the moribund hamsters in the acute phase showed gross jaundice, pulmonary hemorrhage, and renal hemorrhage, mimicking the severe form of human leptospirosis (Weil’s disease). Next, infection experiments were carried out using *Leptospira* strain UP-MMC-SM stored for 1, 2, 3, 4, or 7 months (decline phase) after less than three passages in Korthof’s medium. All the hamsters infected with 1 × 10^4^ of live leptospires stored for 1 or 2 months since the last in vivo passage became moribund in the acute phase, whereas 60–80% of the hamsters infected with the same number of live leptospires stored for 3, 4 or 7 months survived the acute phase (Fig 1B). This suggested that the pathogenicity of the leptospires decreased as the storage period became longer, even in the low passaged strains. All the surviving hamsters were MAT-positive at 15 dpi, confirming that they had been infected with *Leptospira* (Fig 2). Leptospires were recovered from the kidneys of 86.7% (13/15) of the surviving hamsters on 16, 22, 28, and 56 dpi (Fig 2). No leptospires were recovered from the blood, lungs, livers, or spleens of surviving hamsters on 28 and 56 dpi. Thus, most of the hamsters subcutaneously infected with the highly pathogenic *Leptospira interrogans* strain UP-MMC-SM developed fatal acute leptospirosis, but some hamsters recovered from acute sublethal infection and became renal carriers during the chronic phase.

### Comparison of serum creatinine levels and antibody titers by microscopic agglutination test (MAT) between moribund and surviving hamsters in the acute phase of leptospirosis

**Fig 3:**
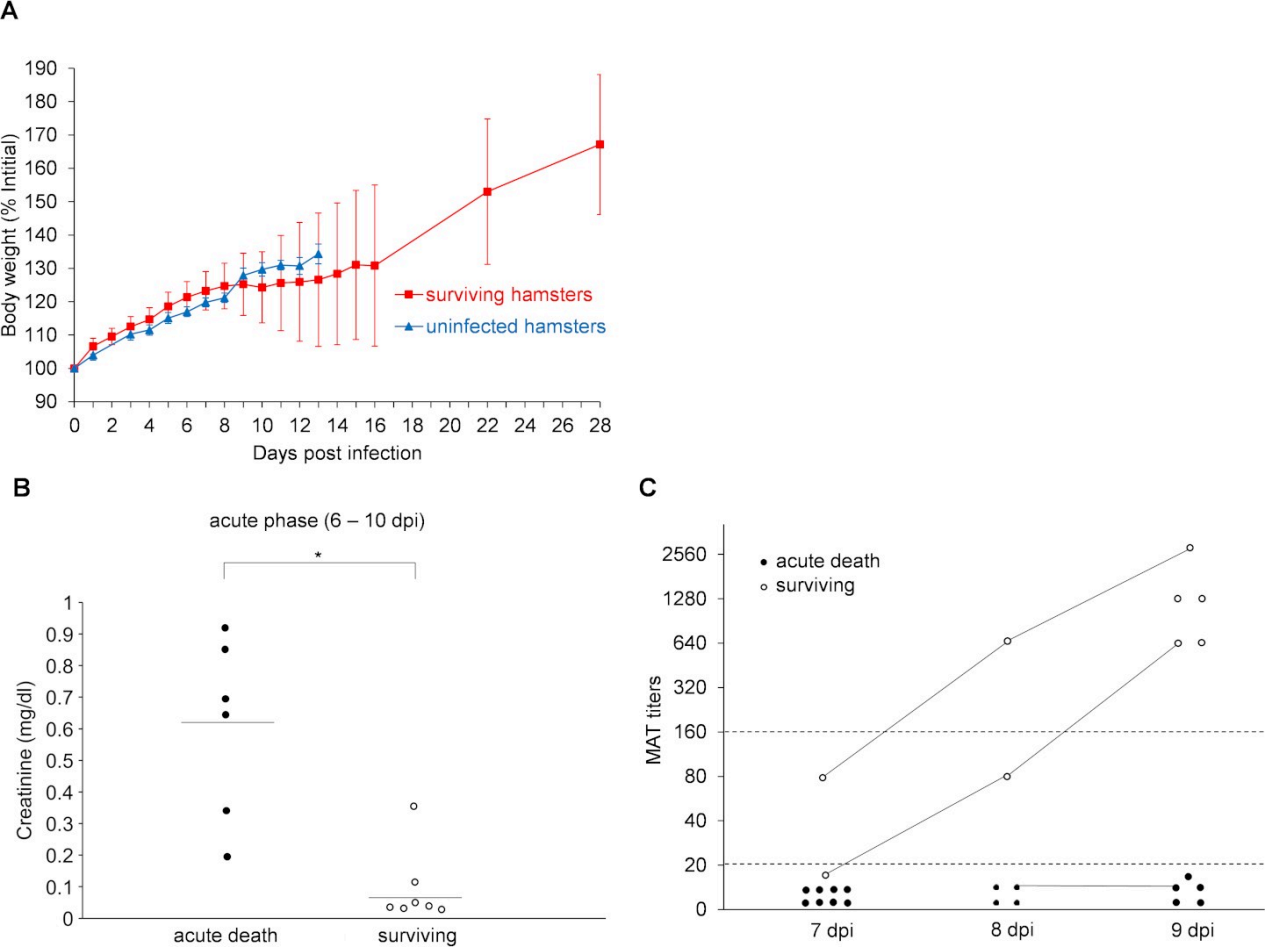
The changes in body weight, serum creatinine levels and antibody titers of moribund and surviving hamsters in the acute phase. (A) The changes in body weight of uninfected control hamsters (blue triangles, n = 8) and surviving hamsters subcutaneously infected with 1 × 10^4^ cells of *L*. *interrogans* serovar Manilae strain UP-MMC-SM (red squares, n = 15) were compared with the body weight on 0 dpi as 100%. Error bars indicate mean ± SD. (B) The serum creatinine levels in the acute phase (6–10 dpi) were compared between hamsters that became moribund in the acute phase (“acute death” black circles, n = 6) and those that survived (“surviving” white circles, n = 7). Moribund hamsters showed higher serum creatinine levels than surviving hamsters. The average value for each group is indicated by a bar. Mann-Whitney U test was used for comparison between the two groups (*p* = 0.006). The significance level was *p* < 0.05. (C) The antibody titers by microscopic agglutination test (MAT) titers in the acute phase (6–10 dpi) were compared between hamsters that became moribund in the acute phase (“acute death” black circles, n = 16) and those that survived (“surviving” white circles, n = 5, 9 samples). The MAT titers of multiple samples obtained from a hamster are connected by a line. *p <0.05 between groups.

The surviving hamsters lost weight with reduced activity during the acute phase, but then recovered in the chronic phase (Fig 3A). The changes in body weight of the surviving hamsters varied from one individual to another. Five out of fifteen hamsters showed no weight loss, activity loss or ruffled fur in the acute phase, but showed positive kidney cultures in the chronic phase. Surviving hamsters began to gain weight at 9 to 15 dpi. To clarify what factors make a difference between life and death for the infected hamsters during the acute phase, serum creatinine levels and MAT titers (anti-Manilae antibody titer) were measured in the hamsters that began to lose weight or became moribund in the acute phase. Serum creatinine levels and antibody titers were compared between the two groups of moribund and surviving hamsters. The serum creatinine levels in the moribund hamsters were significantly higher than those in the surviving hamsters in the acute phase (*p* = 0.006) (Fig 3B). The acute death hamsters had more severe acute renal dysfunction than the surviving hamsters. All of the moribund hamsters (n = 16) were MAT-negative (titer was <160), while all of the surviving hamsters (n = 5) were MAT-positive (titers ranged from 640 to 2,560 on 9 dpi) (Fig 3C). They suggested that the increase in antibody titer in the acute phase was necessary for the hamsters infected with *Leptospira* to survive the acute phase.

### Comparison of the distribution of leptospires and histopathological findings in the kidney of infected hamsters in the acute phase

**Fig 4:**
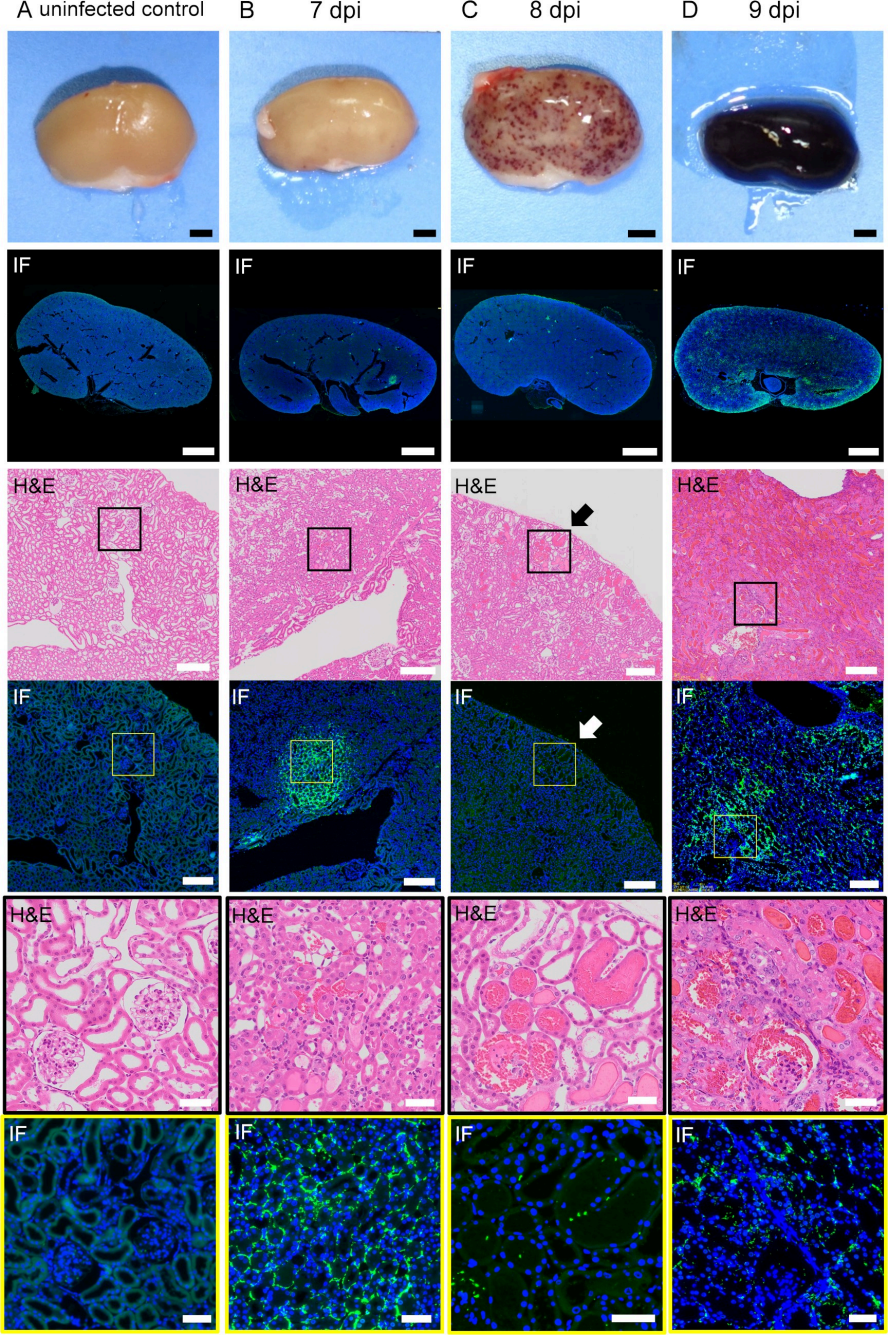
Distribution of leptospires and histopathological findings of infected kidneys in the acute phase. The hamsters were subcutaneously injected with PBS (A) or 1 × 10^4^ cells of *L*. *interrogans* serovar Manilae strain UP-MMC-SM (B, C, D). The left kidneys collected from the moribund hamsters were fixed and sectioned into 4 μm slices, and then stained with immunofluorescent and H&E staining. Comparison of immunofluorescent staining and H&E staining of serial renal sections in the acute phase of infected hamsters. Representative macroscopic images and microscopic images of the serial sections are shown. Leptospires are labeled in green and cell nuclei in blue. The strong green fluorescence at the margin of the kidney is autofluorescence. (A): uninfected control, (B): 7 dpi, (C): 8 dpi, (D): 9 dpi. (B, C, D) Leptospires that colonize the kidneys of hamsters during the acute phase migrate through the interstitium and spread. (C) The section of petechiae on the kidney at 8 dpi was observed by H&E staining. The petechiae on the kidney (arrow) was a part of the hemorrhagic nephron-tubules. The areas boxed by black (third row) or yellow (fourth row) are enlarged in the fifth and sixth rows, respectively. The scale bars are 2 mm in the first and second rows; 200 μm in the third and fourth rows; and 100 μm in the fifth and sixth rows.

The kidneys of the infected hamsters in the acute and chronic phases were serially sectioned at a thickness of 4 μm and were stained with H&E, PAS and fluorescent immunostaining with anti- *Leptospira interrogans* serovar Manilae antibody. Macroscopic findings of the kidneys are shown in Fig 4. Petechiae began to be observed on the kidneys on 7 dpi. The number gradually increased, and the hemorrhage spread throughout the kidneys in the moribund hamsters on 9 dpi. The following histopathological findings were obtained from kidney sections of the hamsters on 7 dpi. H&E staining revealed that the petechial lesions resulted from hemorrhage into the nephron tubules (Fig 4C). Immunofluorescence staining showed that leptospires appeared focally in the cortex on 7dpi (Fig 4B). At higher magnification, the leptospires were located mainly in the interstitium and accumulated continuously along the cortical vessels (Fig 4B). No infiltration of neutrophils was seen around the leptospires (Fig 4B), while local mild inflammatory changes such as congestion and a slight plasma cell infiltration were observed in the cortex (Fig 4B). There were no major abnormalities in the glomerular and tubular epithelium (Fig 4B–4D). Hemorrhage was widespread in the renal tubules, glomeruli, and interstitium in moribund hamsters on 9 dpi (Fig 4D). Leptospires were distributed extensively in the kidney sections (Fig 4D).

### Comparison of the distribution of leptospires and histopathological findings in the kidney of infected hamsters in the chronic phase

**Fig 5:**
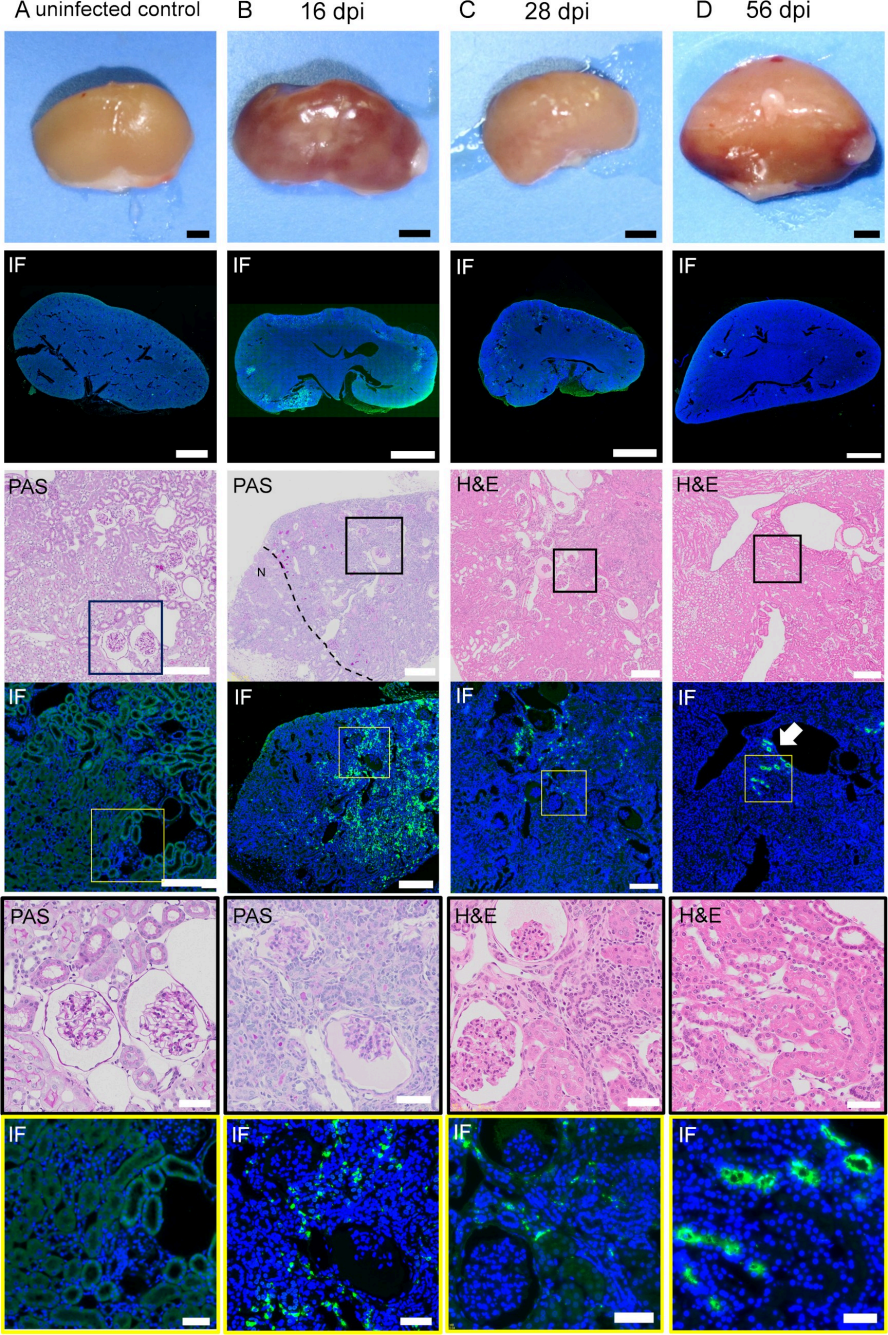
Distribution of leptospires and histopathological findings of infected kidneys in the chronic phase. Hamsters were subcutaneously injected with PBS (A) or 1 × 10^4^ cells of *L*. *interrogans* serovar Manilae strain UP-MMC-SM (B, C, D). The left kidneys collected from the euthanized hamsters were fixed and sectioned into 4 μm slices, and then stained with immunofluorescent, PAS and H&E staining. Comparison was done of immunofluorescent staining and PAS or H&E staining of serial renal sections in the chronic phase (16, 28, 56 dpi) of surviving hamsters. Representative macroscopic images and microscopic images of the serial sections are shown. Leptospires are labeled in green and cell nuclei in blue. The strong green fluorescence at the margin of the kidney is strong autofluorescence. (A): uninfected control, (B): 16 dpi, (C): 28 dpi, (D): 56 dpi. (B) Dotted lines in the PAS staining image of the kidney on 16 dpi indicate the border between the lesion and normal tissue (“N” normal tissue). The distribution of leptospires in the interstitium was substantially consistent with the lesion of tubular atrophy at 16 dpi. (C, D) Arrows (white) in the immunofluorescent staining image of the kidney on 56 dpi indicate the accumulation of leptospires in the renal tubules. Some of the proximal tubules were colonized by *Leptospira* without surrounding inflammatory cell infiltration at 56 dpi. The areas boxed by black (third row) and yellow (fourth row) are enlarged in the fifth and sixth rows, respectively. The scale bars are 2 mm in the first and second rows; 200 μm in the third and fourth rows; and 100 μm in the fifth and sixth rows.

Macroscopic findings of the kidneys are shown in Fig 5. The kidneys of the surviving hamsters showed no hemorrhage, but some kidneys were atrophied with multiple depressed scars on 16 and 28 dpi. The kidney showed irregular contour due to focal tubular atrophy in the surviving hamsters on 16 dpi (Fig 5B). The areas of tubular atrophy were patchy and well demarcated from the normal area (Fig 5B). Moderate infiltrations of lymphocytes and plasma cells were observed in the interstitium and around the blood vessels, but neutrophils were not as prominent as in the acute phase (Fig 5C). The distribution of leptospires in the interstitium was substantially consistent with the lesion of tubular atrophy on 16 dpi (Fig 5B). This finding indicated the possibility that tubular atrophy occurred in the presence of leptospires in the interstitial tissue. The deposition of leptospires in the renal interstitium gradually decreased during the chronic phase (28 dpi), whereas the lesions in the atrophic tubules remained clearly demarcated from normal tissue, as on 16 dpi (Fig 5C). Dense accumulation of leptospires was detected in the lumen of some proximal tubules on 28 and 56 dpi (Fig 5C and 5D). There was no infiltration of lymphocytes or plasma cells even around the proximal tubule where leptospires accumulated (Fig 5D). These results suggested that the distribution of leptospires in the renal interstitium during the acute phase may have affected the extent of the atrophic tubules observed in the chronic phase. This also suggested that leptospires colonized some renal tubules and evaded host immunity there during the chronic phase.

### Comparison of serum creatinine levels between surviving hamsters in the chronic phase and uninfected hamsters

**Fig 6:**
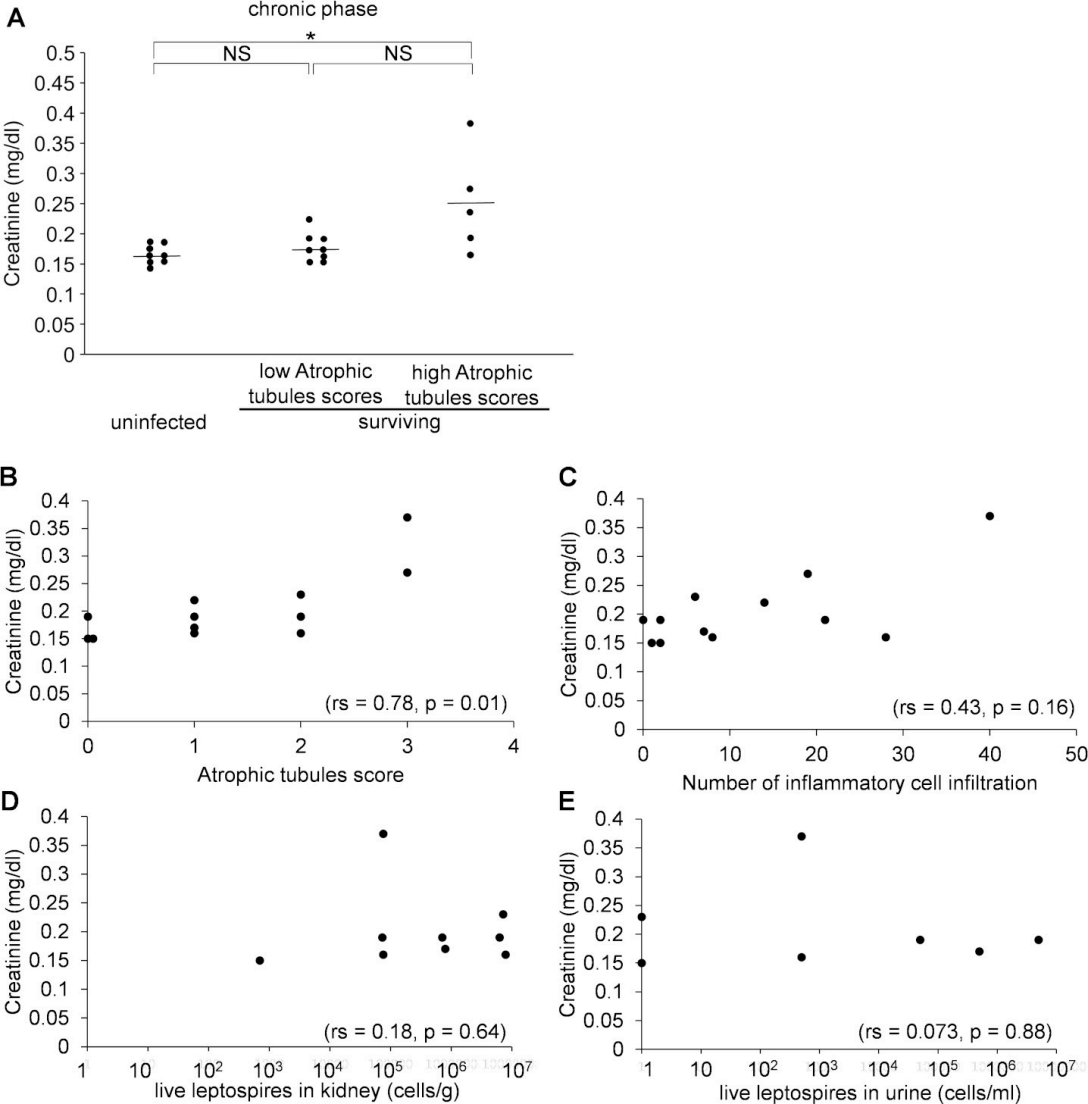
Serum creatinine levels of surviving hamsters in the chronic phase and uninfected hamsters. (A) After infection with *L*. *interrogans* serovar Manilae strain UP-MMC-SM, surviving hamsters were divided into two groups according to the atrophic tubules score. Serum creatinine levels were compared among 3 groups: the high atrophy tubules score group, the low atrophy tubules score group, and uninfected group (n = 5, 8, 8, respectively). The average value for each group is indicated by a bar. The Kruskal-Wallis were used to compare serum creatinine levels among the 3 groups (*p* = 0.035). The significance level was *p* < 0.05. *p<0.05 between groups. NS; not significant. (B-E) Spearman’s rank correlation coefficients for serum creatinine levels and each of the four variables (atrophic tubules score, number of inflammatory cell infiltration, and numbers of leptospires in the kidney and urine) are shown. The number of inflammatory cells infiltration was counted as the spots infiltrated by the inflammatory cells in the whole longitudinal section of the kidney under microscope. The numbers of leptospires in the kidney and urine were measured by the limiting dilution culture method. In surviving hamsters, the serum creatinine level was statistically correlated with the atrophic tubules score. The significance level was p < 0.05. The number of data varies depending on the factor because of the missing value. (B) Scatter plot of serum creatinine and atrophic tubules score (n = 12, rs = 0.78, p = 0.01). (C) Scatter plot of serum creatinine level and the number of spots with inflammatory cell infiltration (n = 12, rs = 0.43, p = 0.16). (D) Scatter plot of serum creatinine and the number of leptospires in the kidney (n = 9, rs = 0.18, p = 0.64). (E) Scatter plot of serum creatinine and urinary leptospires (n = 7, rs = 0.073, p = 0.88).

Serum creatinine levels were measured in the surviving hamsters during the chronic phase to investigate the relationship between leptospirosis and the decline of renal function. The serum creatinine levels of non-infected 6-week-old hamsters were also measured as controls. As there were differences in the degree of tubular atrophy in the kidney sections of the surviving hamsters, the surviving hamsters were divided into 2 groups: a high tubular atrophy group (Atrophic tubules score 2–3) and a low tubular atrophy group (Atrophic tubules score 0–1). Therefore, the serum creatinine levels were compared among three groups: the high tubular atrophy group, the low tubular atrophy group and the uninfected control group. There were significant differences among the 3 groups (*p* = 0.035), especially between the high tubular atrophy group and the uninfected group (Fig 6A). We examined the association between the storage period of injected bacterial cultures and the atrophy tubules scores in the surviving hamsters. The kidneys of hamsters injected with the strains stored for two months or less showed higher atrophy tubules scores in the chronic phase compared to strains stored for 3, 4, and 7 months (S1 Fig). Statistical analysis was not possible due to the small sample size. These results suggested that the renal function of the surviving hamsters was slightly reduced in the chronic phase. Leptospires stored for less than 1 month tended to cause more severe histological changes in the kidneys of surviving hamsters than leptospires stored for over 1 month.

### Factors associated with serum creatinine levels in surviving hamsters

To investigate other factors affecting renal function after acute leptospirosis in addition to the degree of renal tubular atrophy, we also examined the correlation between serum creatinine levels and *Leptospira* burdens or the extent of inflammation in the kidney of surviving hamsters. The numbers of leptospires in the kidney and urine were measured by the limiting dilution culture method, and used as an index of *Leptospira* burdens. The number of spots infiltrated by inflammatory cells in the whole longitudinal section of the kidney was counted and used as an index of the extent of inflammation. The relationship between the serum creatinine level and atrophic tubules score, the number of spots with inflammatory cell infiltration, and the number of leptospires in the kidney or urine are shown by a scatter plot in Fig 6B–6D. The degree of atrophic tubules in the kidney sections correlated statistically with the serum creatinine level in the chronic phase (rs = 0.78, p = 0.01). None of the other factors showed any correlation with the creatinine level (the number of spots with inflammatory cell infiltration, rs = 0.43, p = 0.16; the number of leptospires in the kidney, rs = 0.18, p = 0.64; urinary leptospires, rs = 0.073, p = 0.88). These results suggested that renal tubular atrophy rather than *Leptospira* burdens or the extent of inflammation in the kidney could determine the severity of renal dysfunction in the chronic phase.

### Pathogenicity examination of leptospires excreted in the urine of surviving hamsters in the chronic phase of leptospirosis

**Fig 7:**
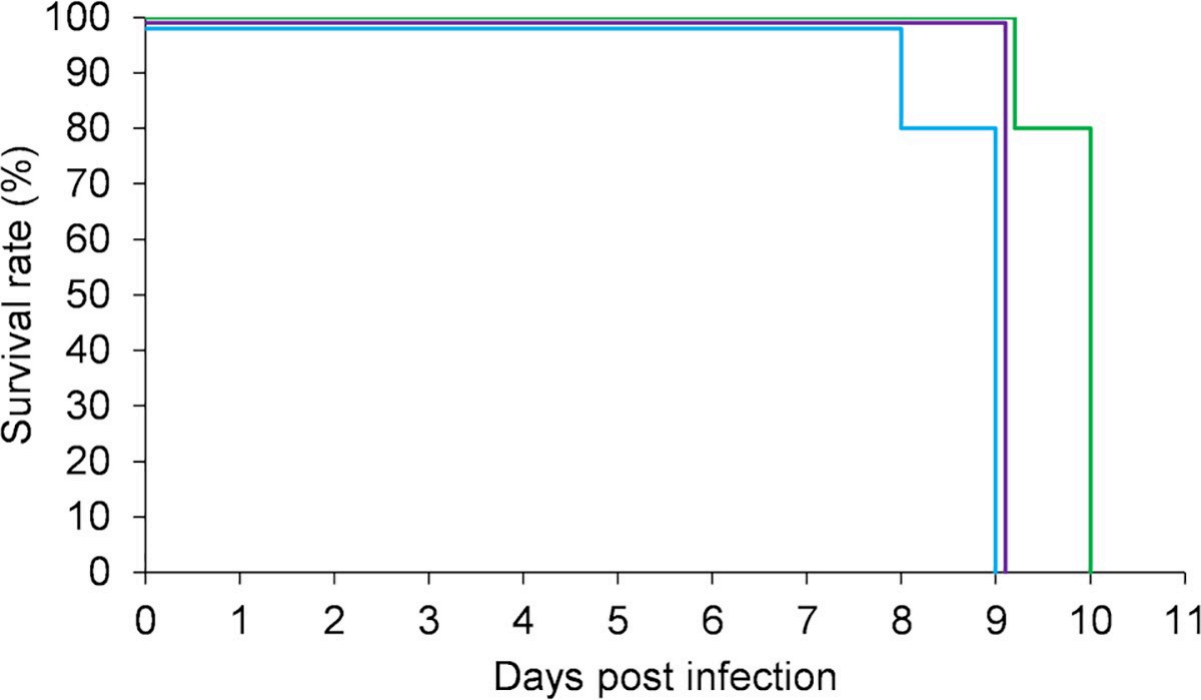
Pathogenicity of leptospires excreted in the urine of surviving hamsters in the chronic phase. Leptospires were isolated from 28 dpi urine of 3 surviving hamsters. Survival curves of hamsters subcutaneously infected with 1 × 10^4^ cells of 3 isolates are shown (n = 5 in each group). Leptospires isolated from the urine of surviving hamsters had high pathogenicity.

A pathogenicity experiment was conducted to investigate whether *Leptospira* excreted in urine from a chronically infected hamster can cause acute infection and death in another hamster. The survival curves of hamsters subcutaneously infected with 1 × 10^4^ leptospires isolated from the urine of three surviving hamsters on 28 dpi are shown in Fig 7. All the *Leptospira* isolated from the urine of three hamsters with chronic leptospirosis had high pathogenicity for hamsters.

## Discussion

In this study using a hamster model, we investigated the dynamics of leptospires and histopathological changes in the kidney, and the extent of renal dysfunction in the acute and chronic phases of leptospirosis. Some hamsters subcutaneously infected with the highly pathogenic *Leptospira* developed fatal acute leptospirosis, while others recovered from acute sublethal infection. We demonstrated that the increase in antibody titers in the acute phase influences the outcome in untreated acute leptospirosis, and suggest that the distribution of leptospires in the kidney during the acute phase (septicemic phase) can influence the decline in renal function during the chronic phase (after immunization).

The strains stored for a long period had reduced pathogenicity and increased the frequency of hamsters surviving the acute phase (Fig 1A and 1B). The UP-MMC-SM strain is known to be highly pathogenic, and our previous study showed that the LD_50_ of the strain was 3.2 × 10^0^ leptospires in subcutaneously infected hamsters [22]. *Leptospira* are often stored in medium for long periods at room temperature in laboratories, but it has been said that pathogenic *Leptospira* may have reduced viability and demonstrate a loss of virulence under these storage conditions [28]. The strain UP-MMC-SM used in this experiment took about 5 days to reach a stationary phase when it was cultured in the Korthof’s medium. Leptospires stored in Korthof’s medium for more than one month go into the decline phase. It is quite possible that nutrient depletion and waste accumulation had a negative effect on *Leptospira* in the present study, and that the host acquired protective humoral immunity before it became lethal. The strains stored for a longer period showed lower atrophic tubules scores in the kidneys of infected hamsters during the chronic phase (S1 Fig). These results suggest that infection of *Leptospira* with reduced pathogenicity may have a relatively small effect on renal function in the chronic phase.

The hamsters that died in the acute phase had more severe renal dysfunction than the surviving hamsters (Fig 3B). It was found that all the hamsters that died in the acute phase were MAT-negative, whereas the surviving hamsters were already MAT-positive when they began to lose weight in the acute phase (Fig 3C). Microscopic agglutination test (MAT) is the gold standard for confirming the diagnosis of leptospirosis, and it can detect antibodies (IgG) for *Leptospira* in serum. These results are consistent with earlier studies showing that MAT serology is insensitive, particularly in early acute-phase specimens [29–31], and patients with fulminant leptospirosis may die before seroconversion occurs [31,32].

Pathologically, tubulointerstitial nephritis is classified into acute and chronic tubulointerstitial nephritis. The former is predominantly composed of acute lesions such as edema and neutrophil infiltration, whereas the latter is predominantly composed of chronic lesions such as interstitial fibrosis and tubular atrophy [33]. In this study, there was no infiltration of inflammatory cells such as neutrophils around the leptospiral antigen in the kidney in the acute phase (Fig 4B–4D). The kidneys in the chronic phase showed peritubular and perivascular lymphocyte and plasma cell infiltration, atrophic tubules and interstitial fibrosis, indicating chronic tubulointerstitial nephritis (Fig 5B and 5C). In atrophic tubules, the entire tubular epithelial cell is seen to regress, and at this stage the tubular damage is thought to be irreversible [34]. The distribution of leptospires in the renal interstitium at 16 dpi in the early chronic phase was mostly consistent with that in the atrophic tubules (Fig 5B). Immunofluorescence staining is a highly sensitive test, but it cannot distinguish between viable and non-viable leptospires [35]. The fluorescence in the kidney interstitium at 16dpi was assumed to be mostly dead leptospires, based on their morphology. Some of the proximal tubules were colonized by *Leptospira* without surrounding inflammatory cell infiltration in the chronic phase (Fig 5D). These results are consistent with earlier studies [36]. This site may be a privileged site for immune evasion. Some hamsters, on the other hand, were chronically colonized but did not have an elevated serum creatinine level. In the recovery period of acute leptospirosis, it may be necessary to distinguish between colonization of the renal proximal tubule lumen and chronic renal damage due to tubulointerstitial nephritis.

Tubular atrophy has been shown to be superior to glomerular pathology as a predictor of CKD progression [37,38]. The surviving hamsters with moderate and high tubular atrophy showed higher serum creatinine levels than the uninfected control hamsters (Fig 6A), and there was a statistical correlation between the chronic-phase serum creatinine levels and the atrophic tubules score (Fig 6B). Unexpectedly, the serum creatinine levels did not correlate with the number of leptospires in the kidney (Fig 6D). This is consistent with a previous report in which there was no correlation between the number of bacteria colonizing the kidneys and the degree of fibrosis in *Leptospira*-infected mice [39]. This experiment assessed the number of viable leptospires by the limiting dilution culture method, which reflected *Leptospira* burdens in the renal tubular lumen during the chronic phase. Colonization of the tubular lumen by *Leptospira* would not affect the fibrosis course. There may be a correlation between *Leptospira* burdens in the acute phase and serum creatinine levels in the chronic phase, considering that hamsters will die if the *Leptospira* burdens in the acute phase are too high. There was no correlation between the number of areas of inflammatory cell infiltration and serum creatinine levels in the chronic phase (Fig 6C), probably because they decreased over time.

In a previous study of long-term renal function outcomes in acute kidney injury caused by leptospirosis, 9% of patients had abnormal renal function consistent with early CKD [12]. A case of leptospirosis that required irreversible dialysis has also been reported [40]. In that case, interstitial fibrosis with tubular atrophy that progressed despite treatment was observed. The patient was already in critical condition requiring hemodialysis at the start of treatment, and the distribution of leptospires in the kidney was presumed to be very wide and the burden was excessive at that time.

From the above, our results suggest that the inflammatory response induced during acute infection, but not during chronic infection, plays a major role in *Leptospira* nephropathy in hamsters. Comparison of models of *Leptospira* infection in resistant and susceptible hosts suggests the importance of the inflammatory response in influencing the outcome of disease. Overexpression of anti-inflammatory IL-10 was faster and at higher levels in resistant mice than in hamsters [41]. Expression of TNF-α, IL-1α, and IL-10 were significantly higher in lethally infected hamsters compared to survivors [42]. TLR2-dependent upregulation of IL-10 induced by *Leptospira* reduces the pathogenesis of *Leptospira* nephropathy in hamsters [43].

*Leptospira* excreted in urine from chronically infected hamsters caused acute infection and death in other hamsters (Fig 7). Asymptomatic renal colonization of leptospires in a region of high disease transmission is common, including among people without serological or clinical evidence of recent infection [7]. Although Human-to-human transmission has been rarely documented [44], further work is required about the effect of chronic renal colonization with *Leptospira* in susceptible animals.

This experiment had some limitations. The results of this experiment may be specific to the highly pathogenic serovar Manilae, and we did not investigate other serovars. In our previous study, we found that similar and varied findings were seen in the various macroscopic and microscopic lesions in hamsters caused by leptospires belonging to the same species but different serovars [24]. All the moribund hamsters had pulmonary hemorrhage, which may have affected the outcome. In this infection experiment, the bacterial condition of the strain was different in each infected hamster due to the difference in storage period. Further studies are needed to understand the influence of long-term storage strains on infection. There are no data on normal serum creatinine levels in hamsters, and it is not clear to what extent the increase in serum creatinine level in this study indicates renal damage.

**Fig 8:**
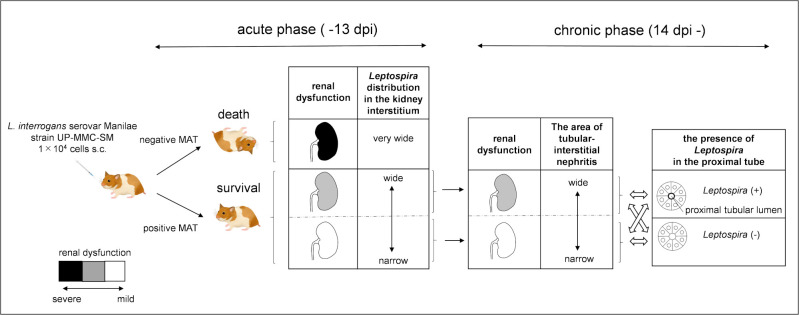
Hamster model of acute and chronic leptospirosis with subcutaneous infection. The color scale of the longitudinal section of the kidney indicates the degree of renal dysfunction. Subcutaneous infection with pathogenic leptospires could cause acute death in hamsters, whereas recovery from acute infection led to chronic leptospirosis. If antibody production is initiated during the acute phase when leptospires colonize the kidney and proliferated there, the growth of leptospires is suppressed and hamsters survive. On the other hand, if antibody production is delayed, leptospires become overloaded and hamsters die. *Leptospira* distribution in the kidney of surviving hamsters was expected to be narrower than that of dead hamsters in the acute phase. The distribution of leptospires in the kidney during the acute phase may affect the chronic renal damage due to tubulointerstitial nephritis. Some hamsters showed leptospiral colonization in the renal proximal tubule lumen during the chronic phase, which was not correlated with elevated serum creatinine levels. The arrows (black) show the passage of time. The double-headed arrow (white) shows the relationship between renal dysfunction and the presence of *Leptospira* in the proximal tube.

The results of this study are summarized in Fig 8. In summary, subcutaneous infection with pathogenic leptospires could cause acute death in hamsters, whereas recovery from the acute infection led to chronic leptospirosis. Our results suggest that the renal distribution of leptospires during the acute phase probably affected the extent of tubular atrophy, leading to CKD. It is possible that leptospirosis is one of the causes of unexplained renal dysfunction in tropical and subtropical regions.

## Supporting information

S1 Fig Storage period and atrophic tubules score. The atrophic tubules scores were compared between hamsters injected with *Leptospira* of storage period for less than 1 month (n = 4) and storage period for over 1 month (n = 8). The average value for each group is indicated by a bar.
(TIF)
