# Supplementary figures and images for: Correlation between renal distribution of leptospires during the acute phase and chronic renal dysfunction in a hamster model of infection with Leptospira interrogans
Source: PLoS Negl Trop Dis. 2021 Jun 18;15(6):e0009410. doi: 10.1371/journal.pntd.0009410 (PMC8213162; doi:10.1371/journal.pntd.0009410)

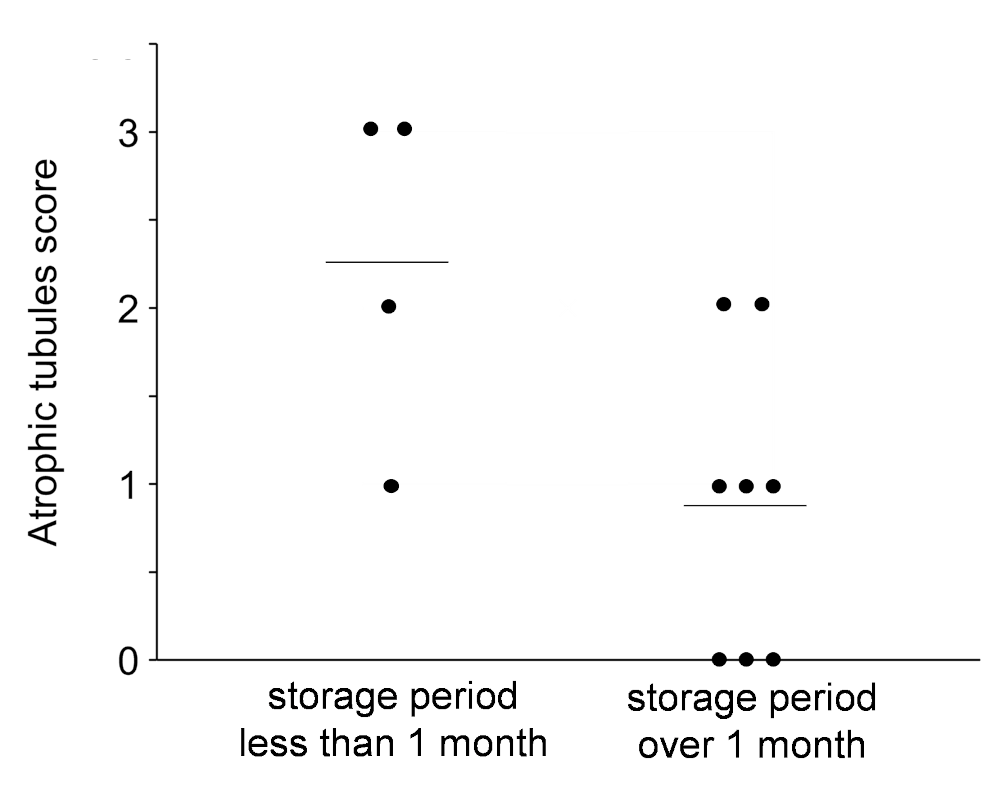

Supplement: S1 Fig — The atrophic tubules scores were compared between hamsters injected with Leptospira of storage period for less than 1 month (n = 4) and storage period for over 1 month (n = 8). The average value for each group is indicated by a bar. (TIF) [file pntd.0009410.s001.tif]
